# The contribution of parity to ethnic differences in mothers' body mass index in the Netherlands: A Blinder-Oaxaca decomposition approach

**DOI:** 10.1016/j.pmedr.2023.102484

**Published:** 2023-10-24

**Authors:** Enise Çayci, Charifa Zemouri, Thijs van den Broek

**Affiliations:** a Erasmus School of Health Policy & Management, Erasmus University Rotterdam, Postbus 1738, 3000 DR Rotterdam, the Netherlands; b Athena Institute, Vrije Universiteit Amsterdam, De Boelelaan 1085, 1081 HV Amsterdam, the Netherlands; c Zemouri Public Health Research & Consultancy, Amsterdam, the Netherlands

**Keywords:** Parity, Body mass index, Ethnicity, Lifestyle, Mediation analysis, Public health

## Abstract

Women of Turkish and Moroccan origin in the Netherlands are relatively likely to have an unhealthy bodyweight. This study sheds light on how ethnic differences in parity, i.e., the number of times a female carried pregnancies to a viable gestational age, contribute to body mass index (BMI) differences between Turkish-born and Moroccan-born mothers aged 35 + and their native Dutch counterparts. We applied a Blinder-Oaxaca decomposition approach to pooled data from four migrant surveys based on national probability samples (n = 2,532). Unlike conventional mediation analyses, the Blinder-Oaxaca approach recognizes that the association between parity and bodyweight may vary across different groups. Our results indicated that Turkish-born and Moroccan-born mothers in the Netherlands had more children and a higher BMI than native Dutch mothers. Regression analyses moreover showed that the parity-BMI gradient was steeper for Turkish-born mothers than for native Dutch mothers. Decomposition using the Blinder-Oaxaca approach indicated that the higher number of children of Turkish-born and Moroccan-born mothers compared to native Dutch mothers contributed substantially to the higher mean BMI in the former groups. The steeper parity-BMI gradient in Turkish-born mothers further amplified the contribution of parity to the higher mean BMI of Turkish-born mothers as compared to native Dutch mothers. Future research is needed to assess to which extent the steep parity-BMI gradient in Turkish-born mothers can be explained by relatively strong barriers to a healthy lifestyle that Turkish-born mothers of a larger number of children may face due to a relatively strongly gendered division of household and childrearing tasks.

## 1 Introduction

Excess bodyweight accounts for almost 4 % of the total burden of disease in the Netherlands (RIVM, 2018). Almost half of adult women in the country are overweight (Statistics Netherlands, 2022a). Moreover, there are substantial variations in bodyweight between ethnic groups (Dagevos and Dagevos, 2008, Derksen et al., 2022, Snijder et al., 2017). Among people of Turkish and Moroccan origin – two of the largest non-Western migrant groups in the Netherlands – it is much more common to have an unhealthy bodyweight than among native Dutch individuals without a migration background (Ibid.).

The current study sheds light on the extent to which ethnic differences in mothers’ bodyweight in the Netherlands are attributable to differences in parity, i.e., the number of times a female carried pregnancies to a viable gestational age. Given the well-documented long-term effects of childbearing on women’s bodyweight (Bastian et al., 2005, Gunderson and Abrams, 2000, Van den Broek and Fleischmann, 2021) and the considerably higher fertility rates of women of Turkish and Moroccan origin compared to native Dutch counterparts (Garssen and Nicolaas, 2008), parity may be expected to play a substantial role in shaping bodyweight differences between mothers of Turkish and Moroccan origin and native Dutch mothers.

We use pooled survey data and employ a Blinder-Oaxaca decomposition approach to estimate the contribution of parity to ethnic differences in mothers' body mass index (BMI) in the Netherlands. Unlike conventional mediation analyses, this approach recognizes that the relationship between parity and bodyweight may vary across ethnic groups. As explained in more detail later, there are reasons to suspect heterogeneity in the effects of parity on mothers’ bodyweight, which, in turn, may mitigate or amplify the contribution of parity to the higher bodyweight of Turkish-born and Moroccan-born mothers compared to native Dutch mothers.

## 2 Background

Having a larger number of children is a known causal risk factor for overweight and obesity in parous women (Van den Broek and Fleischmann, 2021). Multiple mechanisms plausibly underlie this causal association. Raised progesterone levels during pregnancy lead to bodyfat accumulation, particularly during the first and second trimesters of the gestation period (Gunderson and Abrams, 2000), and excessive gestational weight gain is associated with persistently raised bodyweight (Nehring et al., 2011). Having more children may also have a long-term impact on bodyweight via persistent lifestyle changes. It comes with more constraints and responsibilities that make it challenging to maintain an active and healthy lifestyle (Eyler et al., 2002, Grundy and Read, 2015, Kravdal et al., 2020, Van den Broek, 2021), and that, consequently, may result in bodyweight gain (Umberson et al., 2011).

Parity differs notably between ethnic groups in the Netherlands. Although fertility differences have become smaller over the last decades, Moroccan-born and Turkish-born women in the Netherlands still give birth to a greater number of children than their native Dutch counterparts (Garssen and Nicolaas, 2008, Nicolaas and Van Roon, 2020). These differences reflect the family size preferences of the respective ethnic groups (De Valk, 2013). Given the impact of parity on mothers’ bodyweight, ethnic differences in parity may be expected to contribute to bodyweight disparities between groups of mothers with different ethnic backgrounds.

There are reasons to suspect that the long-term implications of having a larger number of children on mothers’ bodyweight may differ between ethnic groups. Women of Turkish or Moroccan origin are relatively often overweight or obese at the beginning of a pregnancy (Bahadoer et al., 2015), which, in turn, is associated with lower gestational weight gain (Santos et al., 2018). Possibly for this reason, they are at a lower risk of excessive gestational weight gain than native Dutch women (Bahadoer et al., 2015; cf. Gaillard et al., 2013). This lower risk may mitigate the parity-bodyweight gradient for Turkish-born and Moroccan-born women, and, consequently, the contribution of parity to ethnic differences in mothers’ bodyweight. On the other hand, the degree to which having more children poses barriers to a healthy lifestyle for mothers is plausibly contingent on the extent to which they can share the caring responsibilities for children with partners, and, compared to their native Dutch counterparts, people of Turkish and Moroccan origin in the Netherlands tend to have more conservative views about how women and men should divide household and childrearing tasks (Dagevos et al., 2016, Van de Vijver, 2007). A more unequal division of such tasks may imply that a greater number of children raises even stronger barriers to a healthy and active lifestyle for Turkish-born and Moroccan-born mothers than for native Dutch mothers. This may strengthen the positive association between number of children and bodyweight among the former groups, and, in turn, amplify the contribution of parity to ethnic differences in mothers’ bodyweight.

## 3 Data and methods

### 3.1 Sample

We use pooled data from four Dutch migrant surveys based on national probability samples: the baseline wave of the Netherlands Longitudinal Lifecourse Study (NELLS) and the 2011, 2015 and 2020 editions of the Survey Integration Minorities (SIM).

NELLS is a survey dataset with an oversample of ethnic minorities, designed to provide insights into social cohesion, values, and social inequalities (Tolsma et al., 2014).. Baseline data collection took place between 2008 and 2010. The sample was drawn in a two-stage procedure. First, 35 municipalities were selected semi-randomly. Second, participants between 15 and 45 years were randomly selected from these municipalities’ population registers. Data were mainly collected via face-to-face interviews and selected information was collected with an additional self-completion questionnaire.

The SIM2011 (Korte and Dagevos, 2011), SIM2015 (Andriessen and Kappelhof, 2016) and SIM2020 (Dagevos and Kappelhof, 2022) surveys were designed to monitor the structural and socio-cultural position of migrants in the Netherlands. The data for SIM2011, SIM2015 and SIM2020 were collected between November 2010 and May 2011, between January 2015 and July 2015, and between March 2020 and January 2021, respectively. In addition to data from native Dutch people without a migration background and people of Turkish or Moroccan origin, data were also collected from people of Surinamese and Dutch Caribbean origin, and, in some editions, from people of Polish, Somali or Iranian origin. For each edition of SIM, random samples per origin group were drawn from the Dutch municipal registry. SIM data were partly collected via face-to-face interviews and partly via web surveys. Among a small fraction of SIM2011 respondents, data were furthermore collected via telephone interviews. SIM2020 respondents were given the extra option to participate via video interviews.

Response rates among people of Turkish origin ranged from 35 % in SIM2020 to 54 % in SIM2011, and response rates among people of Moroccan origin ranged from 28 % in SIM2020 to 50 % in SIM2011. Response rates among native Dutch people were somewhat higher, ranging from 47 % in SIM2020 to 60 % in NELLS Wave 1.

**Fig. 1:**
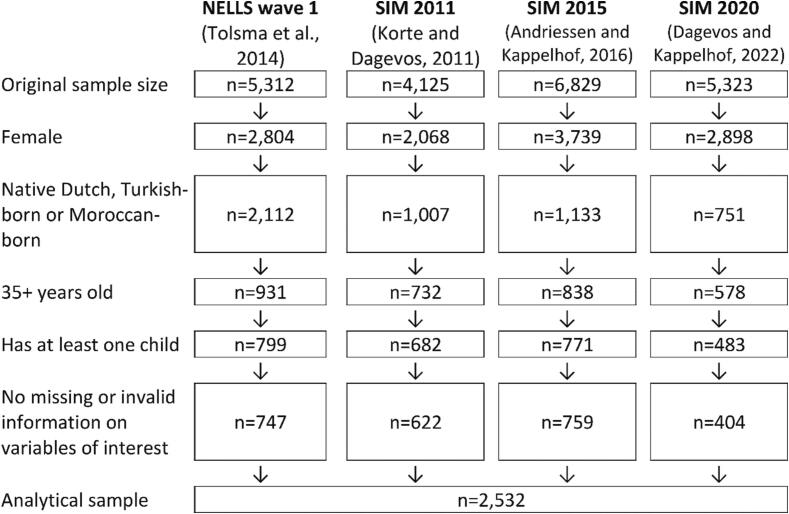
Flow chart of sample selection.

Fig. 1 provides an overview of our study’s inclusion procedure. Given our focus, we restricted the sample to women who had at least one child and were Turkish-born, Moroccan-born or native Dutch. We furthermore only considered respondents aged 35 and older, because only modest increases in numbers of children may be expected beyond this age (Garssen and Nicolaas, 2008). Finally, respondents with missing or implausible (see subsection on measures) values on variables of interest were dropped. The final analytical sample contained 2,532 respondents (646 Turkish-born; 583 Moroccan-born; 1,303 native Dutch). Supplied analytical weights were used to account for selective person non-response. For each of the four surveys, these weights corrected for sex, age and municipality characteristics per ethnic group. Weights in the SIM surveys also took other demographic characteristics (e.g., civil status) into account. To facilitate the pooling of weighted data from multiple sources, the analytical weights were recalibrated so that the average weight in each ethnic group * data source combination was 1.

### 3.2 Measures

BMI was calculated by dividing respondents’ self-reported bodyweight in kilograms by their self-reported height in meters squared (kg/m^2^). We flagged reported bodyweights lower than 25 kg or greater than 250 kg, reported heights lower than 100 cm or greater than 240 cm, or BMI scores lower than 14 kg/m^2^ or greater than 55 kg/m^2^ as implausible (cf. Flegal et al., 2019, van den Broek and Fleischmann, 2022), and excluded respondents with such scores.

The central explanatory variable was self-reported number of children. This variable was log-transformed to account for the substantially positively skewed distribution. Several controls were included to account for potential confounding. Four age categories were distinguished: 35–44 years old, 45–54 years old, 55–64 years old, and 65 + years old. A dichotomous variable distinguished between respondents who had a partner at the time of the interview and their counterparts without a partner. Educational attainment was captured with a categorical variable that distinguished between respondents with lower secondary education or less, higher secondary or lower tertiary education, or higher tertiary education (i.e., at least a bachelor’s degree or equivalent). A dichotomous variable distinguishing between respondents with and without paid employment at the time of the interview was also included. A final categorical variable distinguished the four data sources used.

### 3.3 Analytical strategy

Our analytical approach consisted of three steps. First, we provided simple descriptive statistics of group differences in BMI and parity. Second, we estimated linear regression models of BMI stratified by ethnic group, as denoted in equation (1):

(1) $$Y_{i}^{g}=\beta_{0}^{g}+\sum_{j=1}^{k}\beta_{j}^{g}x_{\mathrm{ji}}^{g}+\varepsilon_{i}$$

Here, outcome $Y_{i}^{g}$ refers to BMI for person *i* in ethnic group *g*. It was regressed on a range of *k* explanatory variables *x_j_* that included number of children and the set of controls described above. $\beta_{0}^{g}$ refers to the intercept and $\varepsilon_{i}$ captures the individual error term. T-tests were performed to assess whether the coefficient estimates for number of children differed systematically between ethnic groups.

Third, we adopted a Blinder-Oaxaca decomposition approach (Rahimi and Hashemi Nazari, 2021) to estimate how differences in both the levels and the coefficients of the explanatory variables contribute jointly to ethnic differences in mothers’ BMI. Extending on equation (1), the mean BMI for group *g* can be denoted as:

(2) $${\overset{¯}{Y}}^{g}=\beta_{0}^{g}+\sum_{j=1}^{k}\beta_{j}^{g}{\overset{¯}{x}}_{j}^{g}$$

The difference in BMI means between groups *g* = 1 and *g* = 2 can then be denoted as:

(3) $$\Delta \overset{¯}{Y}=\sum_{j=1}^{k}\beta_{j}^{2}\left({\overset{¯}{x}}_{j}^{1}-{\overset{¯}{x}}_{j}^{2}\right)+\sum_{j=1}^{k}{\overset{¯}{x}}_{j}^{2}\left(\beta_{j}^{1}-\beta_{j}^{2}\right)+\sum_{j=1}^{k}\left({\overset{¯}{x}}_{j}^{1}-{\overset{¯}{x}}_{j}^{2}\right)\left(\beta_{j}^{1}-\beta_{j}^{2}\right)+\left(\beta_{0}^{1}-\beta_{0}^{2}\right)$$

This difference consists of four components: an endowment component, a coefficients component, an interaction component, and a component for basic differences. The endowment component refers to the products of the group differences in the means on the explanatory variables multiplied by the non-reference group’s coefficients for the respective explanatory variables. Here, it captures the extent to which ethnic differences in mother’s mean BMIs are attributable to group differences in the mean numbers of children and the mean scores on the other explanatory variables.

The coefficients component consists of the products of the non-reference group’s means on the explanatory variables multiplied by the group differences in the coefficient estimates of the respective variables. Here, it captures the extent to which ethnic differences in mother’s mean BMIs are attributable to group differences in coefficient estimates for number of children and the other explanatory variables.

The interactions component refers to the products of the group differences in the means on the explanatory variables multiplied by the group differences in the coefficients of the respective variables. This component accounts for the simultaneous effect of endowments and coefficients. The extent to which group differences in the outcome of interest can be attributed to group differences in both differences in levels and differences in coefficients of the modelled explanatory variables is captured with the sum of the endowment component, the coefficients component and the interaction component. Finally, the basic differences component refers to the difference between groups in the intercepts. It captures the part of the group difference in mean BMI attributable neither to group differences in levels of the modelled explanatory variables, nor to group differences in the coefficient estimates of these variables.

All analyses were performed in Stata 17.1. We used Jann’s (2008) oaxaca-package for the Blinder-Oaxaca decomposition. The delta method was used to produce the standard errors for the various components of the ethnic differences in mothers’ bodyweight.

## 4 Results

### 4.1 Descriptive statistics

Descriptive statistics are presented in Table 1. The mean BMI was approximately 3 kg/m^2^ higher for the Turkish-born and Moroccan-born mothers than for their native Dutch counterparts. These BMI differences were statistically significant. The number of children was furthermore markedly and statistically significantly greater for Turkish-born and, particularly, Moroccan-born mothers than for native Dutch mothers. There were also statistically significant compositional differences between the three groups considered regarding age, partner status, educational attainment, and employment status.

**Table 1:** Descriptive statistics of Turkish-born, Moroccan-born and native Dutch mothers in the Netherlands.

	Turkish-born			Moroccan-born			native Dutch			Turkish-born vs. native Dutch	Moroccan-born vs. native Dutch
	Mean / %	(SD)		Mean / %	(SD)		Mean / %	(SD)		Test of difference	Test of difference
BMI	28.5	(5.1)		28.1	(4.6)		25.3	(4.2)		*F*(1, 1947) = 212.9, *p* <.001	*F*(1, 1884) = 167.0, *p* <.001
Number of children	2.9	(1.3)		3.7	(1.9)		2.3	(1.0)		*F*(1, 1947) = 131.3, *p* <.001	*F*(1, 1884) = 445.8, *p* <.001
Number of children (log)	1.0	(0.4)		1.2	(0.5)		0.8	(0.4)		*F*(1, 1947) = 120.0, *p* <.001	*F*(1, 1884) = 357.7, *p* <.001
Age:										χ^2^(3, n = 1949) = 35.6, *p* <.001	χ^2^(3, n = 1886) = 42.3, *p* <.001
35–44 years old	48.8			54.7			43.9				
45–54 years old	27.9			23.9			20.1				
55–64 years old	11.6			12.1			13.4				
65 + years old	11.7			9.3			22.7				
Has partner	72.7			76.0			81.2			χ^2^(1, n = 1949) = 14.7, *p* <.001	χ^2^(1, n = 1886) = 5.8, *p* <.05
Educational attainment:										χ^2^(2, n = 1949) = 201.4, *p* <.001	χ^2^(2, n = 1886) = 185.4, *p* <.001
Low	71.9			70.9			35.6				
Mid	19.1			20.0			35.8				
High	9.1			9.0			28.6				
In paid employment	32.3			32.3			62.1			χ^2^(1, n = 1949) = 132.2, *p* <.001	χ^2^(1, n = 1886) = 131.4, *p* <.001
Data source:										χ^2^(3, n = 1949) = 34.1, *p* <.001	χ^2^(3, n = 1886) = 41.3, *p* <.001
NELLS Wave 1	23.8			22.7			35.3				
SIM 2011	29.9			27.1			20.8				
SIM 2015	31.6			36.4			26.3				
SIM 2020	14.7			13.7			17.6				
Number of respondents	646			583			1,303				

Notes: Data are from NELLS Wave 1, SIM2011, SIM2015, and SIM2020; Data are weighted; SD: standard deviation; log: after natural log transformation.

### 4.2 Linear regression analyses

Results of the regression analyses of BMI are presented in Table 2. Given the current study’s focus, we will only elaborate on the coefficient estimates of number of children here. After accounting for the other variables in the model, having more children was associated with a higher BMI in the models for Turkish-born and Moroccan-born mothers. In the model for native Dutch mothers, this association was not statistically significant. T-tests indicated that the association between number of children and BMI was significantly stronger for Turkish-born mothers than for native Dutch mothers (Δ*b* = 1.178, 95 % CI: 0.146 to 2.210, *p* <.05). Although the coefficient estimate of number of children was also considerably larger in the Moroccan origin group than in the native Dutch group, this difference was not statistically significant at the conventional alpha level of α = 0.05 (Δ*b* = 0.808, 95 % CI: −0.126 to 1.740, *p* =.09).

**Table 2:** Results of linear regression analyses predicting body mass index of Turkish-born, Moroccan-born and native Dutch mothers in the Netherlands.

	Turkish-born			Moroccan-born			native Dutch	
	B	(95 %CI)		B	(95 %CI)		B	(95 %CI)
Number of children (log)	1.625***	[0.697,2.552]		1.254**	[0.480,2.027]		0.447	[-0.110,1.004]
Age:								
35–44 years old	Ref.			Ref.			Ref.	
45–54 years old	1.223*	[0.273,2.174]		1.555**	[0.619,2.490]		0.087	[-0.614,0.788]
55–64 years old	1.719*	[0.376,3.061]		1.873**	[0.631,3.115]		0.690	[-0.176,1.556]
65 + years old	2.780***	[1.364,4.195]		0.526	[-0.942,1.993]		−0.497	[-1.425,0.430]
Has partner	−0.131	[-0.974,0.712]		−0.119	[-0.989,0.751]		0.106	[-0.502,0.715]
Educational attainment:								
Low	Ref.			Ref.			Ref.	
Mid	−0.217	[-1.228,0.794]		−0.763	[-1.747,0.221]		−1.299***	[-1.867,-0.732]
High	−1.691*	[-3.127,-0.256]		−0.994	[-2.366,0.377]		−2.339***	[-2.950,-1.728]
In paid employment	−1.344**	[-2.247,-0.442]		−1.267**	[-2.124,-0.410]		−0.752*	[-1.379,-0.124]
Data source:								
NELLS Wave 1	Ref.			Ref.			Ref.	
SIM 2011	−0.092	[-1.174,0.989]		−0.111	[-1.185,0.963]		−0.284	[-1.051,0.482]
SIM 2015	1.162*	[0.052,2.272]		−0.710	[-1.741,0.322]		0.482	[-0.249,1.213]
SIM 2020	0.925	[-0.451,2.302]		**−1.037**	[-2.384,0.310]		0.899*	[0.071,1.727]
Intercept	26.293***	[24.933,27.652]		27.157***	[25.805,28.509]		26.266***	[25.314,27.220]
R^2^	0.173			0.115			0.074	
Number of respondents	646			583			1,303	

Notes: Data are from NELLS Wave 1, SIM2011, SIM2015, and SIM2020; Data are weighted; B: Coefficient estimate; CI: Confidence interval; Coefficient estimates in bold differ significantly (*p* <.05) from the corresponding coefficients for the native Dutch group without a migration background;

* *p* <.05; ** *p* <.01; *** *p* <.001.

**Fig. 2:**
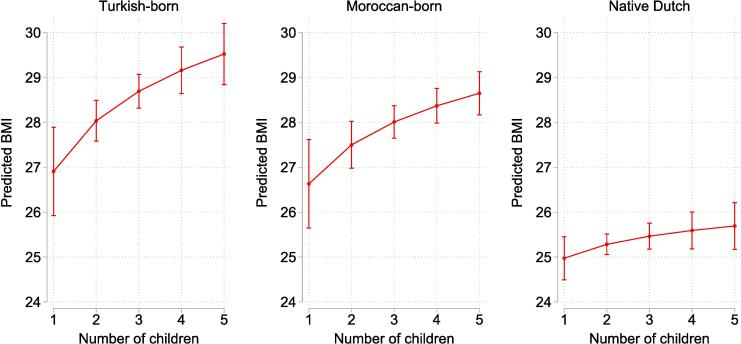
Adjusted predictions of body mass index of mother in the Netherlands by number children and ethnic group.

The log-transformation of our parity variable may make it challenging to grasp the magnitude of the estimated parity-BMI gradients for the three groups. Therefore, Fig. 2 shows adjusted predictions representing the predicted BMI-scores for mothers in particular ethnic groups with one to five children, whereby all other explanatory variables are fixed at the group specific means. The figure clearly shows the steeper parity-BMI gradient for Turkish-born mothers than for native Dutch mothers.

### 4.3 Blinder-Oaxaca decomposition

**Fig. 3:**
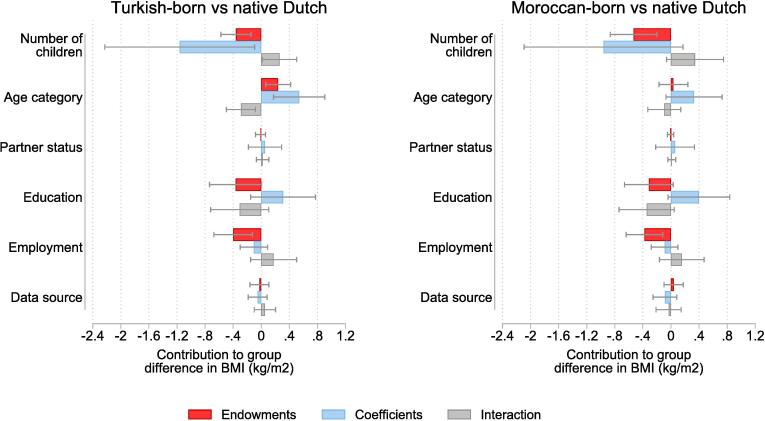
Results of Blinder-Oaxaca decomposition of ethnic differences in mothers’ body mass index in the Netherlands.

Fig. 3 shows the estimated contributions of parity and the covariates considered to BMI-differences between Turkish-born and Moroccan-born mothers and native Dutch mothers. These estimates were obtained using the Blinder-Oaxaca decomposition approach. Again, we will only elaborate on the contributions of number of children here, given the current study’s focus.

The endowment components of the explanatory variables are presented in red. Statistically significant negative endowment components associated with number of children were found for both the mean BMI differences between Turkish-born mothers and native Dutch mothers (*b* = -0.360, 95 % CI: −0.576 to −0.145, *p* <.001) and the mean the BMI differences between Moroccan-born mothers and native Dutch mothers (*b* = -0.532, 95 % CI: −0.865 to −0.199, *p* <.001). In other words, our models predict that the mean BMIs of Turkish-born and Moroccan-born mothers would, with all other factors being equal, be approximately 0.4 kg/m^2^ and 0.5 kg/m^2^ lower, respectively, if they were to have similar numbers of children as their native Dutch counterparts. This corresponds with approximately 11 % and 19 % of the extent to which the mean BMIs of Turkish-born and of Moroccan-born mothers, respectively, exceed the mean BMI of native Dutch mothers.

The coefficients components of the explanatory variables are shown in blue. We found statistically significant evidence for a negative coefficient component associated with number of children when decomposing the mean BMI differences between Turkish-born mothers and native Dutch mothers (*b* = -1.162, 95 % CI: −2.229 to −0.096, *p* <.05). This means that the predicted mean BMI of Turkish-born mothers would, with all other factors being equal, be approximately 1.1 kg/m^2^ lower if the coefficient estimate for number of children in this group would be similar to the coefficient estimate in the native Dutch group. This would correspond with approximately 36 % of the difference in mean BMI between Turkish-born mothers and native Dutch mothers. No statistically significant coefficient component associated with number of children was found for the difference in mean BMI between Moroccan-born mothers and native Dutch mothers (*b* = -0.960, 95 % CI: −2.092 to 0.172, *p* =.10).

The interaction components of the various explanatory variables are presented in grey. The interaction with number of children was statistically significant for the difference in mean BMI between Moroccan-born mothers and native Dutch mothers (*b* = 0.261, 95 % CI: 0.017 to 0.506, *p* <.05). Note that this component’s contribution to the difference in mean BMI between Moroccan-born mothers and native Dutch mothers was positive, whereas the contributions of the endowments and coefficients components were negative. This is because the interaction component accounts for potential simultaneous effects of group differences in endowments and coefficients. In other words, the extent to which the predicted mean BMI of Turkish mothers would be lower if they were to have similar numbers of children as their native Dutch counterparts and if at the same time the coefficient estimate for number of children were similar for them as for native Dutch women is approximately 0.3 kg/m^2^ smaller than the sum of the predicted declines in BMI in the scenario where only numbers of children were similar (endowment component only) and in the scenario where only coefficient estimates were similar (coefficients component only). No statistically significant interaction component associated with number of children was found for the BMI difference between Moroccan-born mothers and native Dutch mothers (*b* = 0.343, 95 % CI: −0.063 to 0.748, *p* =.10).

## 5 Discussion

In line with earlier work (e.g., Garssen and Nicolaas, 2008, Snijder et al., 2017), this study showed that Turkish-born and Moroccan-born mothers in the Netherlands had more children and a considerably higher BMI than their native Dutch counterparts. Regression analyses moreover showed that the parity-BMI gradient was steeper for Turkish-born mothers than for native Dutch mothers. Decomposition using the Blinder-Oaxaca approach indicated that the higher number of children of Turkish-born and Moroccan-born mothers compared to native Dutch mothers contributed substantially to the higher mean BMI in the former groups. The steeper parity-BMI gradient in Turkish-born mothers moreover amplified the contribution of parity to the higher mean BMI of Turkish-born mothers as compared to native Dutch mothers.

Several limitations of the current study should be considered. First, our analyses were cross-sectional, whereby it was impossible to account for possible reverse causation. The estimated impact of parity on bodyweight presented here may be conservative, as obesity can disrupt female fertility (Silvestris et al., 2018).

Secondly, our BMI measure was derived from self-reports of bodyweight and height. Self-reported BMI may differ from BMI based on objectively measured bodyweight and height (Gugushvili and Jarosz, 2019, Krul et al., 2011). Dijkshoorn et al. (2011) noted a stronger downward bias in self-reported BMI among women of Turkish and Moroccan origin than among native Dutch women, which could be explained by the higher objectively measured BMI of women of Turkish and Moroccan origin and the stronger discrepancy between self-reported and objectively measured BMI among people with a higher objectively measured BMI. This suggests that BMI differences between Turkish-born and Moroccan-born mothers and their native Dutch counterparts as well as the contribution of parity to these differences may be underestimated in the current study. Future research using objective BMI measures is called for to avoid underestimation due to selective downward reporting bias.

Finally, we used the reported number of children as a measure for parity. Given that parity refers to the number of times a female carried pregnancies to a viable gestational age, women’s number of children may be lower than their parity if they had stillbirths. Twin births or multiple births can, on the other hand, result in numbers of children that are higher than women’s parity. Note that the impossibility to account for stillbirths nor multiple births in the current study is plausibly only a minor source of bias, because over the last decades, on average approximately 14 per 1,000 live births were multiple births in the Netherlands, and there were on average slightly less than 4 stillbirths with a gestational age of 28 weeks or more for every 1,000 live births (Statistics Netherlands, 2022b).

Despite these limitations, the current study illustrates the usefulness of the Blinder-Oaxaca decomposition approach – which has traditionally mainly been applied in economics – for public health research (cf. Rahimi and Hashemi Nazari, 2021). In prior work, conventional mediation analyses have been performed to assess whether ethnic differences in bodyweight are partly mediated by demographic factors, such as parity (e.g., Bacong and Sohn, 2021, Sohn and Bacong, 2021). The implicit assumption underlying such analyses is that associations between the demographic factors considered and bodyweight do not differ between the groups compared. The analyses presented here provide an example of a case where this assumption is violated, as the parity-bodyweight gradient was systematically steeper for Turkish-born mothers than for native Dutch mothers. Under such conditions, a Blinder-Oaxaca decomposition approach may be more suitable to estimate the contributions of demographic factors, such as parity, to ethnic differences in bodyweight. Alternatively, researchers could adopt a causal mediation analysis approach based on the counterfactual framework. This approach can be applied to any mediation model, including models with interactions between an independent variable, e.g., ethnic group, and mediating variables (Valente et al., 2020).

Substantively, our results underscored the importance of parity as a contributor to mothers’ bodyweight to the extent that differences between ethnic groups in parity contribute meaningly to the substantial ethnic differences in the mean BMIs of mothers in the Netherlands. The findings on Turkish-born mothers moreover provide an example of how a steeper parity-BMI gradient can amplify the contribution of high parity to the extent to which the mean BMI in this group exceeded the mean BMI in the native Dutch comparison group. Our finding of a steeper parity-BMI gradient for Turkish-born mothers compared to native Dutch counterparts was consistent with the theoretical notion that the degree to which having more children poses barriers to a healthy lifestyle for mothers is contingent on the extent to which they can share caring responsibilities with partners. However, data limitations made it impossible for us to test whether the level of inequality in the way women and men share childrearing tasks indeed moderated the parity-BMI gradient. Future research on the moderating impact of the gendered division of childrearing tasks on the parity-BMI gradient is called for, as such research can inform policymakers and practitioners about the degree to which the combination of larger family sizes and unequal divisions of household and childrearing tasks constitutes a catalyst for health inequalities that calls for intervention.

Ethics statement

This study was assessed and approved by the Research Ethics Review Committee of the Erasmus School of Health Policy & Management at Erasmus University Rotterdam (reference: ETH2122-0726).

## CRediT authorship contribution statement

**Enise Çayci:** Methodology, Formal analysis, Visualization, Writing – original draft, Conceptualization. **Charifa Zemouri:** . **Thijs van den Broek:** Methodology, Formal analysis, Visualization, Writing – original draft, Conceptualization.

## Declaration of Competing Interest

The authors declare that they have no known competing financial interests or personal relationships that could have appeared to influence the work reported in this paper.

## Data Availability

The authors do not have permission to share data.

## References

[b0005] Andriessen, I., Kappelhof, J., 2016. Survey Integratie Migranten 2015. Verantwoording van de opzet en uitvoering van een survey onder Turkse, Marokkaanse, Surinaamse, Antilliaanse, Poolse en Somalische Nederlanders en een autochtoon Nederlandse vergelijkingsgroep. SCP Netherlands Institute for Social Research, The Hague.

[b0010] Bacong A., Sohn H. (2021). Disentangling contributions of demographic, family, and socioeconomic factors on associations of immigration status and health in the United States. J. Epidemiol. Community Health.

[b0015] Bahadoer S., Gaillard R., Felix J.F., Raat H., Renders C.M., Hofman A., Steegers E.A.P., Jaddoe V.W.V. (2015). Ethnic disparities in maternal obesity and weight gain during pregnancy. The Generation R Study. Eur. J. Obstet. Gynecol. Reprod. Biol..

[b0020] Bastian L.A., West N.A., Corcoran C., Munger R.G. (2005). Number of children and the risk of obesity in older women. Prev. Med. (baltim).

[b0025] Dagevos, J., Dagevos, H., 2008. Minderheden meer gewicht. Over overgewicht bij Turken, Marokkanen, Surinamers en Antillianen en het belang van integratiefactoren. SCP Netherlands Institute for Social Research, The Hague.

[b0030] Dagevos J., Andriessen I., Vervoort M., Huijnk W., Andriessen I. (2016). Integratie in Zicht? De Integratie Van Migranten in Nederland Op Acht Terreinen Nader Bekeken.

[b0035] Dagevos J., Kappelhof J. (2022).

[b0040] De Valk H.A.G. (2013). Intergenerational discrepancies in fertility preferences among immigrant and Dutch families. Hist. Fam..

[b0045] Derksen E., Knoops K., Mattijssen L., Van Oers F., Voorrips L., De Mooij M., Dieleman D., Van Houdt K., Matthijssen L. (2022). Integratie En Samenleven 2022.

[b0050] Dijkshoorn H., Ujcic-Voortman J.K., Viet L., Verhoeff A.P., Uitenbroek D.G. (2011). Ethnic variation in validity of the estimated obesity prevalence using self-reported weight and height measurements. BMC Public Health.

[b0055] Eyler A.E., Wilcox S., Matson-Koffman D., Evenson K.R., Sanderson B., Thompson J., Wilbur J., Rohm-Young D. (2002). Correlates of physical activity among women from diverse racial/ethnic groups. J. Womens Health Gend. Based Med..

[b0060] Flegal K.M., Ogden C.L., Fryar C., Afful J., Klein R., Huang D.T. (2019). Comparisons of self-reported and measured height and weight, BMI, and obesity prevalence from national surveys: 1999–2016. Obesity.

[b0065] Gaillard R., Durmuş B., Hofman A., Mackenbach J.P., Steegers E.A.P., Jaddoe V.W.V. (2013). Risk factors and outcomes of maternal obesity and excessive weight gain during pregnancy. Obesity.

[b0070] Garssen J., Nicolaas H. (2008). Fertility of Turkish and Moroccan women in the Netherlands. Demogr. Res..

[b0075] Grundy E., Read S. (2015). Pathways from fertility history to later life health: Results from analyses of the English Longitudinal Study of Ageing. Demogr. Res..

[b0080] Gugushvili A., Jarosz E. (2019). Inequality, validity of self-reported height, and its implications for BMI estimates: An analysis of randomly selected primary sampling units’ data. Prev. Med. Reports.

[b0085] Gunderson E.P., Abrams B. (2000). Epidemiology of gestational weight gain and body weight changes after pregnancy. Epidemiol. Rev..

[b0090] Jann B. (2008). The Blinder-Oaxaca decomposition for linear regression models. Stata J..

[b0095] Korte K., Dagevos J., Survey Integratie Minderheden, (2011).

[b0100] Kravdal Ø., Tverdal A., Grundy E. (2020). The association between parity, CVD mortality and CVD risk factors among Norwegian women and men. Eur. J. Pub. Health.

[b0105] Krul A.J., Daanen H.A.M., Choi H. (2011). Self-reported and measured weight, height and body mass index (BMI) in Italy, the Netherlands and North America. Eur. J. Pub. Health.

[b0110] Nehring I., Schmoll S., Beyerlein A., Hauner H., von Kries R. (2011). Gestational weight gain and long-term postpartum weight retention: A meta-analysis. Am. J. Clin. Nutr..

[b0115] Nicolaas H., Van Roon D., De Mooij M., Dieleman D., De Regt S. (2020). Jaarraport Integratie 2020.

[b0120] Rahimi E., Hashemi Nazari S.S. (2021). A detailed explanation and graphical representation of the Blinder-Oaxaca decomposition method with its application in health inequalities. Emerg. Themes Epidemiol..

[b0125] Rivm (2018).

[b0130] Santos S., Eekhout I., Voerman E., Gaillard R., Barros H., Charles M.-A., Chatzi L., Chevrier C., Chrousos G.P., Corpeleijn E., Costet N., Crozier S., Doyon M., Eggesbø M., Fantini M.P., Farchi S., Forastiere F., Gagliardi L., Georgiu V., Godfrey K.M., Gori D., Grote V., Hanke W., Hertz-Picciotto I., Heude B., Hivert M.-F., Hryhorczuk D., Huang R.-C., Inskip H., Jusko T.A., Karvonen A.M., Koletzko B., Küpers L.K., Lagström H., Lawlor D.A., Lehmann I., Lopez-Espinosa M.-J., Magnus P., Majewska R., Mäkelä J., Manios Y., McDonald S.W., Mommers M., Morgen C.S., Moschonis G., Murínová Ľ., Newnham J., Nohr E.A., Andersen A.-M.-N., Oken E., Oostvogels A.J.J.M., Pac A., Papadopoulou E., Pekkanen J., Pizzi C., Polanska K., Porta D., Richiardi L., Rifas-Shiman S.L., Roeleveld N., Santa-Marina L., Santos A.C., Smit H.A., Sørensen T.I.A., Standl M., Stanislawski M., Stoltenberg C., Thiering E., Thijs C., Torrent M., Tough S.C., Trnovec T., van Gelder M.M.H.J., van Rossem L., von Berg A., Vrijheid M., Vrijkotte T.G.M., Zvinchuk O., van Buuren S., Jaddoe V.W.V. (2018). Gestational weight gain charts for different body mass index groups for women in Europe, North America, and Oceania. BMC Med..

[b0135] Silvestris E., de Pergola G., Rosania R., Loverro G. (2018). Obesity as disruptor of the female fertility. Reprod. Biol. Endocrinol..

[b0140] Snijder M.B., Galenkamp H., Prins M., Derks E.M., Peters R.J.G., Zwinderman A.H., Stronks K. (2017). Cohort profile: The Healthy Life in an Urban Setting (HELIUS) study in Amsterdam, The Netherlands. BMJ Open.

[b0145] Sohn H., Bacong A.M. (2021). Selection, experience, and disadvantage: Examining sources of health inequalities among naturalized US citizens. SSM - Popul. Heal..

[b0150] Statistics Netherlands (2022).

[b0155] Statistics Netherlands, 2022b. Geboorte; kerncijfers. [WWW Document]. URL https://opendata.cbs.nl/#/CBS/nl/dataset/37422ned/table?dl=90D6A.

[b0160] Tolsma, J., Kraaykamp, G., De Graaf, P.M., Kalmijn, M., Monden, C.W.S., 2014. The NEtherlands Longitudinal Lifecourse Study (NELLS, Panel). Radboud University Nijmegen, Tilburg University & University of Amsterdam.

[b0165] Umberson D., Liu H., Mirowsky J., Reczek C. (2011). Parenthood and trajectories of change in body weight over the life course. Soc. Sci. Med..

[b0170] Valente M.J., Rijnhart J.J.M., Smyth H.L., Muniz F.B., MacKinnon D.P. (2020). Causal mediation programs in R, Mplus, SAS, SPSS, and Stata. Struct. Equ. Model. A Multidiscip. J..

[b0175] Van de Vijver F.J.R. (2007). Cultural and gender differences in gender-role beliefs, sharing household task and child-care responsibilities, and well-being among immigrants and majority members in the Netherlands. Sex Roles.

[b0180] Van den Broek T. (2021). Early-life circumstances, health behavior profiles, and later-life health in Great Britain. J. Aging Health.

[b0185] Van den Broek T., Fleischmann M. (2021). The causal effect of number of children on later-life overweight and obesity in parous women. An instrumental variable study. Prev. Med. Reports.

[b0190] van den Broek T., Fleischmann M. (2022). Gender differences in bodyweight change following COVID-19 lockdown measures in the Netherlands: A prospective longitudinal study. BMJ Open.

